# Association between skilled maternal healthcare and postpartum contraceptive use in Ethiopia

**DOI:** 10.1186/s12884-018-1790-5

**Published:** 2018-05-16

**Authors:** Gizachew Assefa Tessema, Tensae Tadesse Mekonnen, Zelalem Birhanu Mengesha, Katherine Tumlinson

**Affiliations:** 1 Department of Reproductive Health, Institute of Public Health, University of Gondar, Gondar, Ethiopia; 2 School of Public Health, The University of Adelaide, Adelaide, Australia; 3 Department of Midwifery, Tseda Health Science College, Gondar, Ethiopia; 4 Centre for Health Research, Western Sydney University, Sydney, Australia; 5 Department of Maternal and Child Health, Gillings School of Global Public Health, University of North Carolina at Chapel Hill, Chapel Hill, USA

**Keywords:** Postpartum, Family planning, Maternal healthcare, Ethiopia

## Abstract

**Background:**

The postpartum period provides an important opportunity to address unmet need for contraception and reduce short birth intervals. This study aims to assess the association between skilled maternal healthcare and postpartum contraceptive use in Ethiopia.

**Methods:**

Data for this analysis come from the 2011 to 2016 Ethiopian Demographic and Health Surveys (EDHS) and include nearly 5000 married women of reproductive age with a recent birth. Multivariate logistic regression was conducted to investigate the relationship between skilled maternal healthcare and postpartum contraceptive use.

**Results:**

Between rounds of the 2011 and 2016 EDHS, the postpartum contraceptive prevalence increased from 15 to 23% and delivery in public facilities, use of skilled birth assistance, and skilled antenatal care also grew. In both survey rounds, educated women had approximately twice the odds of postpartum contraceptive use, compared with non-educated women, while an initially significant relationship between wealth and postpartum contraceptive use diminished in significance by 2016. Women with a desire to limit future pregnancy had five to six times the odds of postpartum contraceptive use in both survey rounds, and women in 2016 – unlike those in 2011 – with a desire to *delay* pregnancy were significantly more likely to use contraception (adjusted odds ratio (AOR) = 4.38, 95% CI: 1.46-13.18) compared to women who wanted another child soon. In 2011, no statistically significant associations were found between any maternal healthcare and postpartum contraceptive use. In contrast, in 2016, postpartum contraceptive use was significantly associated with an institutional delivery (AOR = 1.71, 95% confidence interval (CI): 1.12-2.62) and skilled antenatal care (AOR = 2.41, 95% CI: 1.41-4.10). No significant relationship was observed in either survey round between postpartum contraceptive use and skilled delivery or postnatal care.

**Conclusions:**

A comparison of postpartum women in the 2011 and 2016 EDHS reveals increased use of both contraception and skilled maternal healthcare services and improved likelihood of contraceptive use among women with an institutional delivery or antenatal care, perhaps as a result of increased attention to postpartum family planning integration. Additionally, results suggest postpartum women are now using contraception to space future pregnancies, with the potential to help women achieve more optimal birth intervals.

## Background

Approximately 20% of births in low-income countries are to women with fewer than 24 months since their previous birth, resulting in a significant number of birth intervals of short duration [1–3]. The critical public health importance of optimal birth intervals for women and children are well-documented. Birth intervals spanning two to five years are associated with improved child nutrition, reduced likelihood of preterm birth and low birth weight, and – subsequently – increased child survival [4–6]. Optimal child spacing may also reduce the likelihood of abortion, miscarriage, and stillbirth and reduces competition among siblings [1, 7]. In addition to these benefits for children and infants, women with healthy birth intervals are at reduced risk of maternal death and pregnancy-related morbidity [8]. As such, family planning programs have great potential to reduce maternal, child, and infant mortality in developing country settings by helping women achieve healthy birth intervals. Experts estimate nearly one third of all maternal deaths and close to 10 % of deaths to children under five years of age could be averted by eliminating short birth intervals [4, 9].

One key strategy for ensuring optimal birth intervals is the promotion of contraceptive use in the postpartum period [1]. Evidence suggests that the vast majority of women in developing countries wish to avoid pregnancy in the first year following a birth, but unmet need within this vulnerable group is consistently higher than in women outside of the extended postpartum period; some estimates of unmet need in the extended postpartum period are as high as 65% [10–13]. A variety of reasons may contribute to unmet need among postpartum women; yet several studies conducted in countries in Africa and Asia have demonstrated that use of maternal health services, such as pre- and postnatal care and institutional delivery, can enhance postpartum family planning use [14–18]. Therefore, the postpartum period provides a unique and critical opportunity to fulfil unmet need for contraceptives and to reduce the risks of closely spaced pregnancies [13]. Integration of family planning counselling with pre- and postpartum services has the potential to help motivated women achieve adequate birth spacing as well as limit unintended pregnancy. A recent study provides compelling evidence that the provision of information on family planning to women in the postpartum period resulted in significantly increased contraceptive use [19].

Ethiopia has a total population of 95 million people, nearly one quarter of which are women of reproductive age. The catchment area of primary health care facilities (public hospitals, health centres, and health posts) covers nearly 92% of the population [20]. Despite the potential to reach a vast majority of the Ethiopian population, in reality, a much smaller portion of the population utilise skilled maternal healthcare services in Ethiopia: in 2016, 26% of pregnant women delivered in a health facility, 62% of pregnant women received skilled antenatal care, and 17% received skilled postnatal care [21]. The current prevalence of modern contraceptive use in Ethiopia is approximately 25% of all women of reproductive age and 35% of married women of reproductive age; nearly one out of every four married women of reproductive age has an unmet need for family planning [21, 22]. An analysis of birth intervals in 44 low-income countries found 46% of parous women sampled in the 2005 Ethiopia Demographic and Health Survey (EDHS) reported birth intervals of less than two years. In comparison, among the 25 other African countries included in the analysis, the percent of women with a birth interval under two years ranged from 24 to 54% and, when compared with Ethiopia, only four African countries had a larger percentage of women with birth intervals under two years [5].

The 2011 Ethiopian national guidelines for family planning services mandate integration of family planning services with all skilled maternal healthcare services [23]. Government health policies also aim to increase the contraceptive prevalence rate among married women of reproductive age to 55% and to reduce unmet need to 10% by 2020 [24]. One key and promising national strategy being implemented in an effort to achieve these targets is promotion of postpartum contraceptive use through the scale-up of existing postpartum family planning services by expanding the services to district level health facilities and universal access of contraceptive methods in the immediate postpartum period in 2020 [24, 25]. Towards this end, additional information is needed to better understand whether use of skilled maternal healthcare services such as pre- and postnatal care and institutional delivery might impact subsequent postpartum contraceptive use in Ethiopia.

The present study uses large scale national datasets collected in 2011 and 2016 to examine the association between use of skilled maternal healthcare services and subsequent postpartum contraceptive use among married reproductive aged women in Ethiopia, and compares the findings between the two surveys. The findings of this study can inform policies and programs designed to improve postpartum family planning use, reduce unmet need, and reduce the prevalence of short birth intervals among Ethiopian women.

## Methods

### Study design and sample

**Fig. 1:**
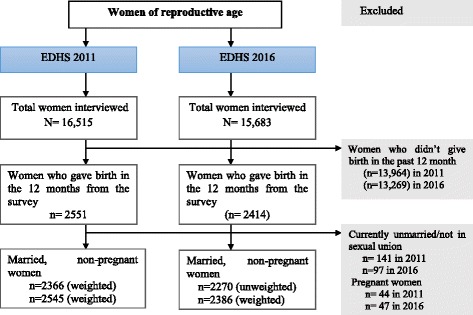
Schematic presentation showing sampling in the 2011 and 2016 Ethiopia Demographic and Health Surveys (EDHS)

The Ethiopian Demographic and Health Survey (EDHS) was first implemented in Ethiopia in 2000 and a total of four national surveys have been conducted as of 2016. The present analysis uses data from the latest two rounds of the EDHS, collected in 2011 and 2016. The EDHS is a cross-sectional survey conducted every five to six years to measure the demographic and health-related characteristics of the population [26]. The EDHS 2011 survey was conducted from December 2010 to June 2011 and includes interviews with a total of 16,515 women of reproductive age [27]. The EDHS 2016 survey was conducted from January to June 2016 and includes a sample of 15,683 women of reproductive age [21]. A stratified, two-stage cluster sampling procedure was used to recruit the nationally representative sample in both surveys. The 11 administrative regions found in Ethiopia were initially stratified into urban and rural areas. Each stratum was subdivided into districts (*woredas),* and *woredas* were then subdivided into *kebeles* (the smallest administrative unit in Ethiopia). Finally each kebele was subdivided into Enumeration Areas (EAs) that served as sampling clusters. A total of 624 EAs were included in EDHS 2011 and 645 EAs were included in EDHS 2016. In the present analysis, we included married/in sexual union, non-pregnant women who gave birth in the 12 months prior to data collection. As a result, a total of 2545 married/in sexual union women in EDHS 2011 and 2386 married/in sexual union women in EDHS 2016 were included in our final analysis (Fig. 1). The analysis was restricted to married/in sexual union women to allow cross-country comparisons of contraceptive prevalence rate (CPR), which is often calculated only for married women and those in sexual union. Additionally, we excluded women who were not married or in sexual union because they are not considered at risk for pregnancy and therefore do not require methods of pregnancy prevention, the main outcome of interest in this analysis.

### Postpartum contraceptive use

In this analysis the dependent variable is a binary variable for postpartum contraceptive use. Postpartum contraceptive use is defined as any self-reported use of a modern contraceptive method (modern methods reported in this sample included: female sterilization, implant, intrauterine device (IUD), injectable, oral contraceptive, emergency contraceptive, lactational amenorrhea method (LAM) or condom) at the time of data collection. Those women not reporting any modern method use and those reporting traditional method use (periodic abstinence or withdrawal) were classified as non-users.

### Skilled maternal healthcare services

We examined the association between postpartum contraceptive use and four key maternal healthcare services, all coded as binary variables: institutional delivery, presence of a skilled birth attendant, skilled antenatal care, and skilled postnatal care. Institutional delivery was classified based on the place of delivery. A woman was defined as having an institutional delivery if she had given birth (the most recent time) at a hospital, health centre, or any private facility; otherwise women were classified as having a home delivery. Skilled attendance at birth was based on who attended the delivery irrespective of the place of delivery. Those women whose most recent birth was attended by a professional (doctor, midwife or nurse, health officer) possessing midwifery skills were classified as having skilled attendance at birth. Skilled antenatal care included any woman receiving one or more episodes of antenatal care by a skilled provider. Skilled postnatal care was based on health check-ups by skilled professionals (doctor, midwife or nurse, health officer) for women who delivered in a health facility or at home within the first six weeks of the participant’s most recent delivery.

### Demographic and reproductive and maternal health variables

Demographic, reproductive health and other factors were also examined as potential covariates in this analysis. Demographic variables include age, rural versus urban residence, region, religion, participant’s education, partner’s education, employment status, wealth, and parity. Reproductive health and other factors include birth order, concordant family planning preferences, fertility preferences, women’s decision-making, contraceptive knowledge (the ability to mention at least one method), any visit from a family planning worker in the last 12 months, and exposure to family planning media messages (newspaper, radio, television, or mobile SMS text) in the last few months. Women’s decision making was defined by whether a woman reports that she can make decisions by herself or jointly with her partner about the following: decisions about health service utilisation, decisions about household purchases, and decisions to visit family or relatives. Specific categories for these variables are shown in Tables 1, 2, and 3.

**Table 1:** Sociodemographic characteristics in the 2011 and 2016 Ethiopia Demographic and Health Surveys (EDHS), among married/in sexual union women of reproductive age

Variables	EDHS 2011 (*N* = 2545)		EDHS 2016 (*N* = 2386)	
	Frequency	Percent	Frequency	Percent
Age in years				
15-24	833	32.7	745	31.2
25-34	1203	47.3	1194	50.0
35+	509	20.0	447	18.7
Mean age (SD)	27.68(±6.5)		27.63(6.3)	
Place of residence				
Urban	332	13.1	287	12.0
Rural	2213	86.9	2099	88.0
Region				
Tigray	139	5.5	168	7.0
Affar	22	0.9	23	1.0
Amhara	543	21.3	449	18.8
Oromiya	1136	44.6	1041	43.6
Somali	79	3.1	114	4.8
Benishangul-Gumuz	29	1.1	26	1.1
Southern Nation, Nationalities and People Region	525	20.6	488	20.4
Gambela	7	0.3	5	0.2
Harari	6	0.2	6	0.3
Addis Ababa	53	2.1	58	2.4
Dire Dawa	8	0.3	9	0.4
Religion				
Orthodox	908	35.7	792	33.2
Muslim	979	38.5	1042	43.7
Protestant	586	23.0	475	19.9
Other/missing	72	2.8	78	3.3
Highest educational status				
None	1703	66.9	1417	59.4
Primary	745	29.3	755	31.6
Secondary+	97	3.8	215	9.0
Partner highest educational status				
None	1223	48.0	1056	44.3
Primary	1100	43.2	969	40.6
Secondary+	223	8.7	361	15.1
Women occupation				
Not working	1355	53.2	1476	61.9
Working but not paid/paid in kind only	512	20.1	585	24.5
paid with cash	678	26.6	326	13.7
Wealth index				
Low	1160	45.6	1085	45.5
Middle	539	21.2	491	20.6
High	846	33.2	810	33.9

**Table 2:** Reproductive health related characteristics in the 2011 and 2016 Ethiopia Demographic and Health Surveys (EDHS), among married/in sexual union women of reproductive age

Variables	EDHS 2011 (*N* = 2545)		EDHS 2016 (*N* = 2386)	
	Frequency	Percent	Frequency	Percent
Number of living children				
No child	24	0.9	15	0.6
1-2 child	942	37.0	939	39.4
3-4 child	746	29.3	653	27.4
5+	834	32.8	780	32.7
Birth order				
First	393	15.4	480	20.1
2-3 birth	848	33.3	749	31.4
4-5 birth	565	22.2	510	21.4
6+	739	29.0	647	27.1
Family size concordance				
Both want same	982	38.6	998	41.8
Husband wants more	662	26.0	580	24.3
Husbands wants fewer	187	7.4	147	6.1
Do not know/Missing	714	28.1	662	27.7
Fertility preference				
wants soon	151	5.9	187	7.8
wants later	1387	54.5	1298	54.4
wants no more	892	35.1	760	31.9
Undecided	115	4.5	142	5.9
Women’s decision-making^b^				
Yes	1272	50.0	1623	68.0
No	1273	50.0	764	32.0
Knowledge of modern contraceptive				
Yes	2463	96.8	2351	98.5
No	82	3.2	35	1.5
Visited by FP worker in the last 12 months				
Yes	416	16.3	778	32.6
No	2129	83.7	1608	67.4
Exposure to family planning media^a^				
Yes	689	27.1	592	24.8
No	1856	72.9	1794	75.2

^a^Exposure to family planning media was determined by whether a woman reported she has heard/read about family planning from radio/television/newspaper/ mobile SMS. ^b^Women’s decision-making was defined by whether a woman reported that she herself has made decisions (or made a joint decision with her partner) about her own health care, large household purchase, or visiting families/relatives

**Table 3:** Maternal healthcare services related characteristics in the 2011 and 2016 Ethiopia Demographic and Health Surveys (EDHS), among married/in sexual union women of reproductive age

Variables	EDHS 2011 (*N* = 2545)		EDHS 2016 (*N* = 2386)	
	Frequency	Percent	Frequency	Percent
Current contraceptive use				
Yes	378	14.9	553	23.2
No	2167	85.1	1834	76.8
Place of delivery				
Home	2272	89.3	1499	62.8
Health facility	273	10.7	887	37.2
Place of delivery by facility type^a^				
Public facility	234	85.7	864	97.4
Private facility	39	14.3	23	2.6
Who assisted delivery				
No assistance/ assisted by unskilled provider	2221	87.2	1478	62.0
Doctor	29	1.2	99	4.1
Nurse/midwife/HO	295	11.6	809	33.9
Skilled ANC				
Yes	820	32.2	1620	67.8
No	1725	67.8	766	32.7
Number of skilled ANC^b^(*n* = 820)				
One times	117	14.3	126	7.8
2-3 times	356	43.4	677	41.8
4+ times	347	42.3	817	50.4
Skilled PNC use				
Yes	183	7.18	455	19.1
No PNC	2362	92.18	1931	80.9

*ANC* Antenatal care, *PNC* Postnatal care, *HO* Health officer

^a^*n* = calculated from facility delivery, ^b^*n* = calculated from those who received skilled ANC

### Statistical analysis

We used STATA version 14.0 to conduct all the analysis. Descriptive statistics were calculated for all variables described above. Multivariate logistic regression was conducted to estimate adjusted odds ratios. Those demographic and reproductive health variables described above which had a *p*-value of less than 0.2 in the binary logistic regression analysis were included as covariates in our model. During EDHS 2011, some areas were over-sampled in order to have regional estimates. Additionally, non-response was observed in the survey. In order to ensure the actual representativeness of survey results at the national level, all analyses in the present study were weighted. The complete sample weight computation is available with the dataset provided by ICF International.

## Results

### Socio-demographic characteristics

This analysis includes nearly 5000 married/in sexual union women of reproductive age with a birth in the 12 months preceding the survey; 2545 of these women participated in the 2011 EDHS while the remaining 2386 were participants in the 2016 survey. As seen in Table 1, about half of all women (47% in 2011 and 50% in 2016) were between the ages of 25 and 34, with a median age of 28 years. The majority of the respondents (87% in 2011 and 88% in 2016) were rural residents. Oromo and Amhara ethnic groups were the predominant groups in both rounds of the EDHS. With regard to the level of education, the proportion of women who did not have any formal education decreased from 67% in 2011 to 59% in 2016 and the proportion of women who completed secondary education more than doubled between surveys (4% in 2011 to 9% in 2016). Likewise, the proportion of participants whose partners completed secondary education nearly doubled between surveys from 9 to 15%. About 53% of these postpartum women were not working outside the home. When looking at the household wealth, 46% of participants in both surveys were in the poorest group, with no change between surveys in terms of the distribution of wealth.

### Reproductive health characteristics of women in the postpartum period

As shown in Table 2, in both surveys, more than a third of women had between one and two children, more than a fourth had three to four children, and nearly a third had five or more children. For the majority of participants (80 to 85%), their most recent birth was not their first birth, although first-time deliveries increased by about 5 % when comparing the 2011 and 2016 samples. Few changes were seen between surveys in terms of family size concordance, with just about 40% of couples agreeing on ideal family size and approximately one in four women reporting that their partner wants a bigger family than they do. In both surveys, fertility preferences remained approximately stable, with only about six to 8 % of women reporting a desire for another child soon; about one third reported not wanting any more children and more than half reported wishing to delay their next pregnancy. Ability to mention at least one contraceptive method remained nearly universal in both survey rounds and exposure to family planning media messages stayed stable at around 25%. Notably, the percentage of women reporting some degree of decision-making power increased from 50 to 68%. Also of note, the percentage of women who reported a visit by a family planning worker in the preceding 12 months doubled between surveys from 16 to 33%.

### Postpartum contraceptive and skilled maternal healthcare utilisation

**Fig. 2:**
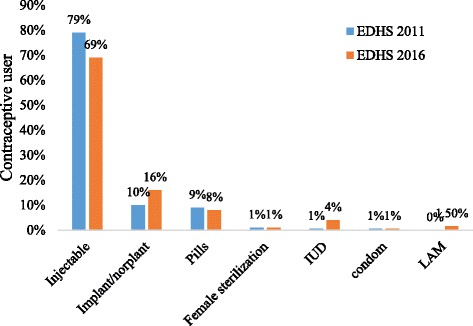
Contraceptive method mix among postpartum women using modern contraceptive methods in 2011(*n* = 378) and 2016 (*n* = 553) in the Ethiopia Demographic and Health Surveys (EDHS)

In the last five years, the prevalence of contraceptive use increased by 53% from 15% in 2011 to 23% in 2016 (Table 3). In terms of the contraceptive method mix in both surveys, injectable contraception was the predominant method (Fig. 2). While there were little to no changes in the contribution to the national method mix by pills, female sterilization, condoms, and LAM, there was a notable change in the contribution of implants (10% in 2011 vs 16% in 2016) and IUD (1% in 2011 vs 4% in 2016) (Fig. 2).

With respect to maternal healthcare, while only about one in ten women reported delivery in a health facility in 2011, more than a third of our 2016 sample reported delivery in a health facility. Use of public facilities became nearly universal within our 2016 sample, as compared to private facility use, among those women not delivering at home. Additionally we observed a threefold increase in the use of skilled assistance during delivery and a twofold increase in the number of women receiving skilled antenatal care, when comparing the two survey samples. We also observed modest increases in the number of women with four or more antenatal visits and skilled postnatal care (Table 3).

### Factors associated with postpartum contraceptive use

In our 2011 sample of postpartum women, two demographic characteristics were strongly associated with postpartum family planning. Women in the highest wealth category had nearly three times the odds of postpartum contraceptive use compared to women in the lowest wealth category. In addition to wealth, education was strongly associated with postpartum contraceptive use in the 2011 sample; those with at least a primary education were nearly twice as likely to use contraception, compared to women with no education. In our 2016 sample of postpartum women, education remains associated with contraceptive use but the magnitude of the association between wealth and family planning use is greatly reduced and the result is only marginally significant (Tables 4 and 5).

**Table 4:** Multivariate logistic regression examining factors associated with postpartum contraceptive use in Ethiopia, 2011 (*n* = 2545)

Variables	PPFP Use		OR (95% CI)			
	No	Yes	COR	95% CI	AOR	95% CI
Wealth index						
Low	1091	69	1		1	
Middle	479	61	2.01	1.16 - 3.50	1.55	0.89 - 2.72
High	597	249	6.61	4.20 - 10.39	2.74***	1.53 - 4.92
Age category (in years)						
15-24	642	191	1		1	
25-34	1055	148	0.47	0.32 - 0.69	0.82	0.51 - 1.31
35+	470	39	0.27	0.17 - 0.46	0.75	0.32 - 1.76
Residence						
Urban	186	147	6.77	4.55-10.07	1.89	0.93 - 3.84
Rural	1981	231	1		1	0.27-1.13
Access to family planning media						
No	1654	203	1		1	
Yes	513	175	2.79	1.94-4.00	1.41	0.91- 2.18
Women educational status						
No education	1564	139	1		1	
Primary	547	198	4.07	2.92 - 5.68	1.83*	1.22 - 2.77
Secondary or above	56	41	8.19	4.15 - 16.17	1.02	0.42 - 2.48
Partner education						
No education	1120	102	1		1	
Primary	893	207	2.54	1.67 - 3.84	1.23	0.73 - 2.06
Secondary or above	154	69	4.92	2.69 - 8.99	0.62	0.27 - 1.40
Women occupation						
Not working	1147	208	1		1	
Working but not paid	463	463	0.59	0.35 - 0.99	0.79	0.46 - 1.36
Working for paid	557	121	1.20	0.83 - 1.74	1.08	0.73 - 1.60
Number of living children						
No child	23	1	1		1	
1-2 child	703	239	8.69	1.89 - 40.03	2.68	0.40 - 17.83
3-4 child	652	93	3.64	0.77 - 17.40	1.35	0.19 - 9.78
5+ child	769	45	1.45	0.30 - 6.95	0.89	0.10 - 7.73
Decision making						
Not participated	1136	136	1		1	
Participated	1031	242	1.95	1.34 - 2.85	1.40	0.90 - 2.16
Fertility Preference						
Wants soon	144	7	1		1	
Wants later	1176	211	3.78	1.18 - 12.10	2.59	0.64 - 10.56
Wants no more	749	143	4.01	1.20 - 13.42	5.90*	1.43 - 24.44
undecided	98	17	3.69	0.93 - 14.52	4.58	1.00 - 21.02
Place of delivery						
Home	2009	264	1		1	
Health facility	158	115	5.53	3.68 - 8.30	0.63	0.18 - 2.19
Skilled attendance at birth						
No	2055	301	1		1	
Yes	112	77	6.13	4.24 - 8.88	2.07	0.69- 6.24
Skilled antenatal care						
No	1564	161	1		1	
Yes	603	217	3.48	2.40 - 5.03	1.16	0.72- 1.89
Skilled postnatal care						
No	2059	303	1		1	
Yes	108	75	4.74	3.02 - 7.45	1.86	0.94- 3.65
Birth order						
First	297	96	1		1	
2-3	653	195	0.93	0.60 - 1.43	1.09	0.61 - 1.94
4-5	515	50	0.31	0.17 - 0.54	0.59	0.25 - 1.41
6+	702	37	0.16	0.08 - 0.30	0.50	0.16 -1.60

*PPFP* Postpartum Family Planning, *COR* Crude Odds Ratio, *AOR* Adjusted Odds Ratio *** *p* < 0.001, *p < 0.05

**Table 5:** Multivariate logistic regression examining factors associated with postpartum contraceptive use in Ethiopia, 2016 (*n* = 2386)

Variables	PPFP Use		OR (95% CI)			
	No	Yes	COR	95% CI	AOR	95% CI
Wealth index						
Low	933	152	1		1	
Middle	383	108	1.72	1.12 - 2.65	1.45	0.90 - 2.36
High	518	292	3.46	2.34 - 5.09	1.53	0.97 - 2.39
Age category (in years)						
15-24	538	208	1		1	
25-34	925	269	0.75	0.55 - 1.03	0.94	0.59 - 1.50
35+	371	76	0.53	0.33 - 0.86	1.18	0.57 - 2.42
Residence						
Urban	136	152	1		1	
Rural	1698	401	0.21	0.14 - 0.31	0.56	0.27-1.13
Access to family planning media						
No	1454	352	1		1	
Yes	380	200	2.17	1.54 - 3.06	0.95	0.64 - 1.40
Women educational status						
No education	1203	213	1		1	
Primary	523	232	2.51	1.79 - 3.50	1.32	0.90 - 1.93
Secondary or above	107	107	5.63	3.58 - 8.85	2.03*	1.09 - 3.78
Partner education						
No education	899	158	1		1	
Primary	710	259	2.08	1.44 - 3.02	1.36	0.95 - 1.96
Secondary or above	225	136	3.44	2.24 - 5.30	0.75	0.41 - 1.37
Number of living children						
No child	8	7	1		1	
1-2 child	640	299	0.56	0.10 - 3.12	0.22	0.04 - 1.30
3-4 child	507	145	0.34	0.06 - 1.82	0.21	0.03 - 1.28
5+ child	678	102	0.18	0.03 - 1.02	0.15	0.02 - 1.00
Visited by FP worker in the past 12 months						
No	1289	319	1		1	
Yes	545	233	1.73	1.24 - 2.40	1.40	0.99 - 1.99
Fertility Preference						
Wants soon	172	15	1		1	
Wants later	955	343	4.23	1.70 - 10.55	4.38**	1.46 - 13.18
Wants no more	589	171	3.42	1.28 - 9.09	5.14**	1.59 - 16.59
undecided	117	24	2.43	0.77 - 7.67	3.44	0.97 - 12.17
Place of delivery						
Home	1289	210	1		1	
Health facility	545	342	3.85	2.81 - 5.23	1.71*	1.12 - 2.62
Skilled attendance at birth						
No	1689	436	1		1	
Yes	144	117	3.15	2.15 - 4.62	1.28	0.81 - 2.01
Skilled antenatal care						
No	706	61	1		1	
Yes	1128	492	5.08	3.01 - 8.46	2.41**	1.41 - 4.10
Skilled postnatal care						
No	1558	373	1		1	
Yes	276	180	2.72	1.96 - 3.75	1.03	0.68 - 1.56
Birth order						
First	321	159	1		1	
2-3	528	221	0.85	0.58 - 1.24	1.02	0.63 - 1.64
4-5	416	94	0.46	0.30 - 0.70	0.83	0.38 - 1.81
6+	569	79	0.49	0.17 - 0.46	0.69	0.25 - 1.94

*PPFP* Postpartum Family Planning, *COR* Crude Odds Ratio, *AOR* Adjusted Odds Ratio ** *p* < 0.01, *p < 0.05

In the 2011 sample of postpartum women, the strongest association with postpartum contraceptive use was seen among women who reported a desire to avoid future pregnancy. Women who wanted no more children had six times the odds of postpartum contraceptive use compared to women who would like to have a child soon. We did not find any relationship between women with a desire to delay pregnancy and contraceptive use in our 2011 sample of women. In the 2016 data, our sample of women also demonstrated a strong likelihood to use contraception in the postpartum period if they preferred no more children; in contrast with the 2011 data however, our 2016 sample of women were also significantly more likely to use contraception if they had a desire to *delay* a future pregnancy (AOR: 4.38, 95% CI: 1.46 – 13.18) (Tables 4 and 5).

Regarding use of skilled maternal healthcare, no statistically significant associations were found in the 2011 sample of women. Women who delivered in a health facility or with a skilled attendant were no more likely than women with home births or those without skilled attendance to use postpartum family planning. Similarly, women with antenatal care visits were no more likely to use postpartum family planning than those without such visits. We found only a marginally significant relationship between postnatal care and postpartum contraceptive use in the 2011 data. In contrast, delivery within a health facility as well as obtaining skilled antenatal care are both significantly associated with postpartum contraceptive use in the 2016 sample, with adjusted odds ratios of 1.71 (95% CI: 1.12-2.62) and 2.41 (95% CI: 1.41-4.10) respectively (Tables 4 and 5).

## Discussion

This study describes the prevalence of postpartum contraceptive use and skilled maternal healthcare among two representative samples (collected in 2011 and 2016) of married/in sexual union women in Ethiopia with a birth in the previous year. Our findings in both rounds are far lower than a finding from similar study in Indonesia (74%) [28]. In our 2011 sample, we found low contraceptive use; only 15% of women were protected from early pregnancy. This is much lower than the overall prevalence of modern method use reported by married women in the 2011 EDHS (approximately 27%), despite the increased health risks to women in the postpartum period [29]. Contraceptive use was much higher (23%) in our 2016 sample of married/in sexual union postpartum women, although still not keeping pace with the overall prevalence of modern method use among all married women of reproductive age in Ethiopia (35% in the 2016 EDHS). Additionally, when compared with the 2011 sample, women in our 2016 sample reported a higher prevalence of facility delivery, greater use of public facilities for delivery, higher prevalence of skilled assistance during delivery (especially nurse and midwife assistance), and a higher prevalence of receiving skilled ante- and postnatal care. In fact, the proportion of women reporting skilled birth assistance or pre- or postnatal care more than doubled and institutional births more than tripled during the five-year period. This growth between 2011 and 2016 in the prevalence of both contraceptive use and skilled maternal healthcare suggests government efforts to increase access to and use of these services are rapidly succeeding. However, the remaining large numbers of women giving birth at home and/or without assistance and without skilled postpartum care indicate the need for these efforts to continue.

In addition to increased uptake of both contraception and skilled maternal healthcare, we also noted a shift in the role played by both wealth and fertility intentions. In the 2011 data, residing in the highest wealth category was strongly associated with an increased likelihood of postpartum contraceptive use. In other words, among women in the 2011 sample, wealthier women were nearly three times more likely to use contraception compared to women in the lowest wealth category. This finding is consistent with studies conducted throughout Africa which have found that wealthier women were more likely to be using contraception compared to their poorer counterparts [17, 18, 30]. Although family planning services in Ethiopia are offered free of charge in public facilities, this finding could reflect indirect costs, such as transportation, which may prohibit access to family planning services. In the 2016 data however, wealth was only marginally significant in terms of its relationship with postpartum contraceptive use, with an adjusted odds ratio of far less magnitude (1.5 compared to 2.7 in 2011). This finding suggests that wealth may play a much smaller role in women’s postpartum contraceptive behaviour compared to five years ago, perhaps as a result of increased outreach efforts; indeed, the proportion of participants reportedly visited by a family planning worker in the 12 months preceding the survey doubled between survey rounds. This finding is a promising indication that the prevalence of contraceptive use is not only rising, but that it is growing in ways that promote greater health equity.

Additionally, we saw in the 2011 data that women who didn’t want any more children were significantly more likely to use family planning, compared to women with a desire to become pregnant again right away. This finding is unsurprising as we would expect greater contraceptive use among women who have already achieved (or possibly surpassed) their desired number of children, particularly when compared with women indicating a desire to have another child soon. However, women in the 2011 sample who wanted to delay (rather than avoid) their next pregnancy were no more likely to use family planning than women who wanted to become pregnant soon. In contrast, in the 2016 sample we see that women with a desire to delay their next pregnancy have more than 4 times the odds of family planning use compared to women who want a pregnancy soon. This suggests that postpartum women in Ethiopia are beginning to use contraception not just to avoid a future pregnancy but also to *delay* their next pregnancy; this increase in the use of contraception for birth spacing represents great promise in government efforts aimed at achieving optimal birth intervals and addressing unmet need for family planning.

Across both rounds of data collection and in agreement with numerous other studies [18, 31, 32], women’s educational status was found to be associated with postpartum contraceptive use. In both samples, women who had at least some primary or secondary education had about twice the odds of contraceptive use as compared to non-educated women. Female education is widely thought to play an important role in fertility [33–35] and these findings support that conclusion.

Finally, this study investigated the association between several key maternal healthcare services and postpartum contraceptive use. Although numerous studies have shown a relationship between the use of some type of maternal healthcare service and postpartum contraceptive utilization, in the 2011 sample of women, these services did not show significant association. While these results may suggest no correlation between skilled maternal healthcare and subsequent postpartum contraceptive use in Ethiopia, we hypothesise two alternative explanations for this finding. First, it is possible that providers were offering family planning information to clients at the time they received one or more types of maternal healthcare services, but the amount or type of information provided may have been insufficient to result in actual contraceptive use. In other words, providers may have done a poor job of discussing information on a variety of methods or may have rushed through the information without verifying that women still recovering from labour and attending to a newborn are fully comprehending their contraceptive options and necessary next steps for obtaining their method of choice. Second, this finding could be the result of poor integration of family planning counselling and services during the provisions of skilled maternal healthcare. Services may be poorly integrated for a variety of reasons. Service providers may be poorly trained on integration protocols or may be simply unable to provide family planning information if experiencing a high case-load of clients. For example, a study in the capital city of Ethiopia showed that nearly two-third of pregnant women accessing antenatal care services did not receive any counselling about contraceptive methods [36]. Further, even those providers who are well-trained may lack sufficient motivation to follow integration protocol unless increased supervision or other accountability mechanisms are in place [37–40].

In contrast to the null findings in our 2011 sample regarding skilled maternal healthcare and postpartum contraceptive use, within our 2016 sample, we found that both delivery within a health care facility and receipt of skilled antenatal care approximately doubled the odds of postpartum contraceptive use. These findings are in line with previous studies investigating the impact of postpartum family planning integration on subsequent contraceptive use [15–19, 31]. The fact that these associations did not exist in the 2011 sample lends some weight to our hypothesis that postpartum family planning messages and services were not well integrated in Ethiopia in 2011 compared to 2016. The new associations emerging in the 2016 data suggest that the Ethiopian ministry of health is succeeding in their demonstrated efforts to integrate contraceptive messages and services during the prenatal period as well as at some point before the patient exits the facility after giving birth. In other words, not only are women having more skilled care before, during, and after delivery, our findings suggest that women may also receive more exposure to family planning services during this critical time; It appears that facilities are capitalising on the convenience of reaching women with family planning messaging while they are already at the facility giving birth.

Importantly, despite remarkable progress in postpartum contraceptive use and increased use of skilled maternal healthcare in Ethiopia, there are still large numbers of women giving births at home, without benefit of skilled antenatal care or skilled delivery, and without skilled postnatal care following their birth. These findings suggest the Ethiopian government would benefit from examining the variety of factors that may be keeping skilled care beyond the reach of large numbers of women, for example the quality of family planning counselling provided during outreach to rural women in their residential homes. Currently, the Ethiopian government has an ambitious plan to achieve nearly universal coverage for ante- and postnatal care and skilled birth attendance [25]. There is much progress remaining in obtaining this goal and the government should continue to prioritize and reinforce efforts to expand and improve maternal healthcare, postpartum contraceptive use, and integration of family planning into all reproductive health services.

This study is not without limitations. First, the data used in this analysis are self-reported and therefore may bias results if participants felt the need to alter their responses based on perceived preferences of the enumerators. Second, while participants report on whether or not they received various types of maternal healthcare services, they did not report on the content of those services and – as we suggest above – it may be the case that not all service providers adhere to national guidelines that mandate family planning integration into reproductive healthcare services. Additionally, it is possible that some women may have started a contraceptive method at some point following their most recent birth but may have discontinued that method prior to being interviewed. In this case, it is possible that some women with some postpartum contraceptive use would have been misclassified as non-users. However, the majority of contraceptive users selected injectable contraception which has a duration of three months following each injection; we would not expect discontinuation rates to be high in the short period of time between contraceptive initiation and the date of interview, particularly among those women who did not immediately initiate contraception following birth and/or those whose last birth preceded the interview date by only a few months. In this regard, for instance, the present analysis shows that nearly two thirds of our participants commence contraceptive six months after delivery. Finally, participants vary in terms of the amount of time since their recent birth, with the time between birth and interview ranging from one to 12 months. Those with fewer months since their recent birth may differ from those with more distant births, and this heterogeneity may warrant further exploration in subsequent analyses.

## Conclusion

In conclusion, the present study revealed that postpartum contraceptive use in Ethiopia is low but increased between 2011 and 2016. Unsurprisingly, educational obtainment and the desire to avoid future pregnancy were positively associated with postpartum contraceptive use in both survey rounds; however 2016 saw a diminished role of wealth as a key correlate of contraceptive use and an emerging association among women with a desire to delay their next pregnancy. Within our 2011 sample, several key maternal healthcare services such as antenatal care and institutional delivery had no association with postpartum contraceptive use, but these null findings became positive associations in the 2016 sample. These results suggest national efforts to strengthen the integration of family planning counselling and services with skilled maternal healthcare services are succeeding, allowing health care facilities to reduce missed opportunities for increasing contraceptive use among this critical population within Ethiopia. Efforts should continue given the large numbers of women in Ethiopia still delivering at home and without the benefit of skilled pre- and postnatal care.

## Abbreviations

AOR: Adjusted odds ratio
CI: Confidence interval
CPC: Carolina Population Center
CPR: Contraceptive prevalence rate
DHS: Demographic and health survey
EA: Enumeration area
EDHS: Ethiopian demographic and health survey
EHNRI: Ethiopian Health Nutrition Research Institute
FP: Family planning
IUD: Intrauterine device
LAM: Lactational amenorrhea method
NICHD: National Institute of Child Health & Human Development
NRERC: National Research Ethics Review Committee
PPFP: Postpartum family planning
